# Evaluating 12 Years of Implementing a Multidisciplinary Specialist Child and Adolescent Obesity Treatment Service: Patient-Level Outcomes

**DOI:** 10.3389/fnut.2022.895091

**Published:** 2022-06-03

**Authors:** Cathy Wyse, Lucinda Case, Órla Walsh, Catherine Shortall, Norah Jordan, Lois McCrea, Grace O'Malley

**Affiliations:** ^1^Obesity Research and Care Group, School of Physiotherapy, Division of Population Health Sciences, RCSI University of Medicine and Health Sciences, Dublin, Ireland; ^2^W28GO Child and Adolescent Obesity Service, Children's Health Ireland at Temple Street, Dublin, Ireland; ^3^Adolescent Medicine and General Paediatrics, Children's Health Ireland at Temple Street, Dublin, Ireland; ^4^Department of Paediatrics, School of Medicine, RCSI University of Medicine and Health Sciences, Dublin, Ireland

**Keywords:** pediatric [MeSH], multidisciplinary, obesity treatment, complex interventions, family-based therapy, personalized treatment

## Abstract

**Introduction:**

Childhood obesity is a chronic disease that requires multidisciplinary and specialist intervention to address its complex pathophysiology, though access to treatment is limited globally. Evaluating the impact of evidence-based interventions implemented in real-world clinical settings is essential, in order to increase the translation of research into practice and enhance child health outcomes. In Ireland, the National Model of Care for Obesity highlighted the need to develop and improve healthcare services for children and adolescents with obesity.

**Aims:**

This study aims to evaluate the impact of a family-based, Tier 3 multi-disciplinary child and adolescent obesity outpatient service (www.w82go.ie) on standardized body mass index (BMI-SDS).

**Methods:**

Following referral by pediatricians, patients were assessed by a pediatric multidisciplinary team (physiotherapist, dietician, and psychologist) and personalized obesity treatment plans were developed. Anthropometric and demographic information were recorded at baseline and final visit. Descriptive statistics were used to explore distribution, central tendency and variation in the demographic data, change in BMI-SDS over time was assessed using a *t*-test, and multiple linear regression analysis was used to investigate the association of demographic factors on the change in BMI-SDS.

**Results:**

The overall mean BMI-SDS reduction across the whole cohort (*n* = 692) was −0.17 (95% CI = −0.20, −0.13; *P* < 0.001). Younger age at admission and longer duration of treatment were associated with greater BMI-SDS reduction but there was no significant association between change in BMI-SDS and any of the other parameters (deprivation score, treatment type, sex, obesity category at admission or presence of comorbid condition).

**Conclusion:**

Engagement in a specialist Tier 3 pediatric obesity service was associated with reductions in BMI-SDS in children and adolescents with obesity.

## Introduction

**Figure 1:**
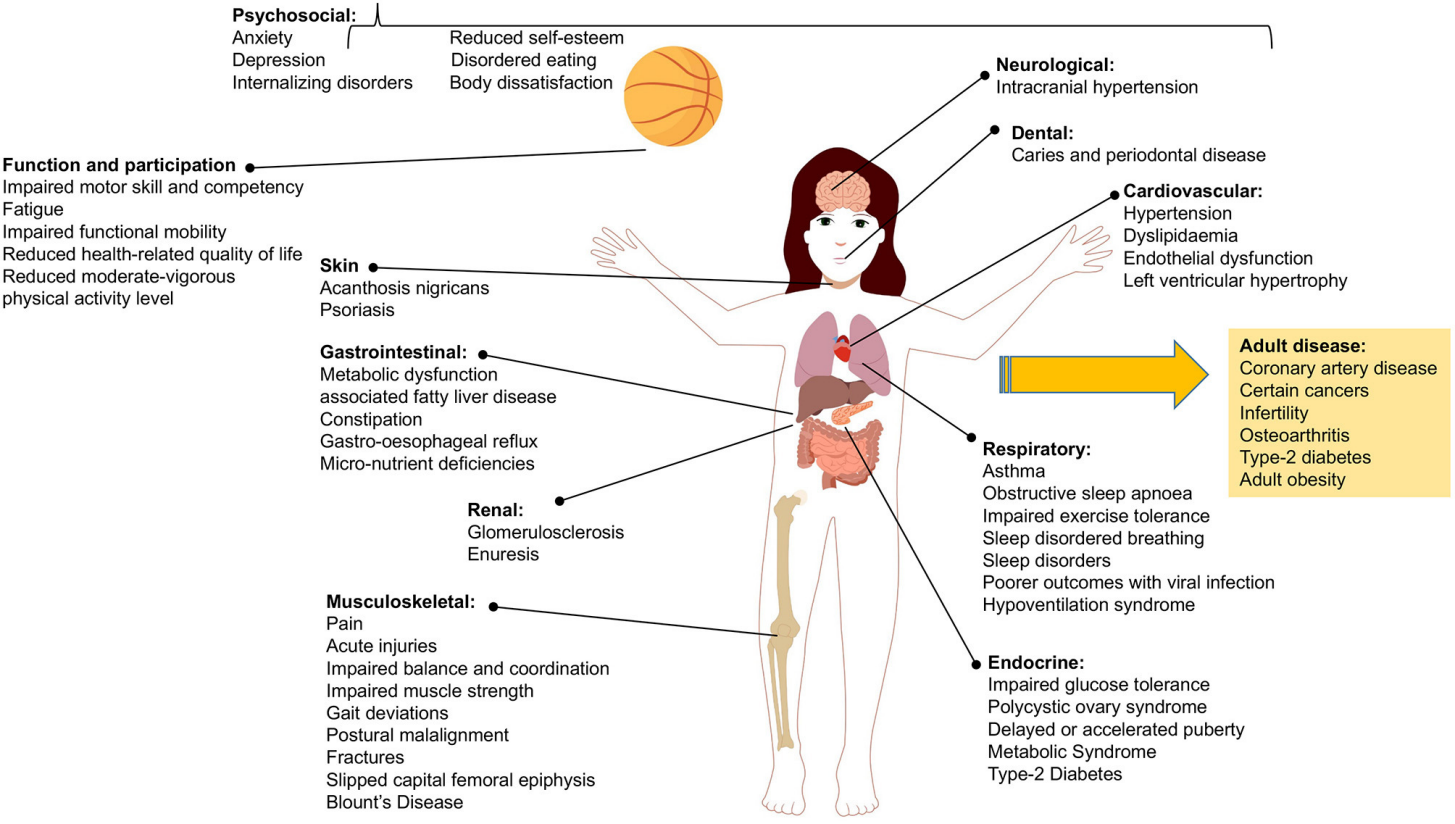
Range of health complications and impairments associated with child and adolescent obesity including those diseases that are of increased risk in adulthood.

Obesity and overweight compromise the health and wellbeing of children worldwide, with global prevalence estimated at 240 million children affected, the majority in low and middle income countries and with greater burden borne by those living in disadvantage (1, 2). Childhood obesity affects children across diverse demographics and environments, often causing challenges to their health and wellbeing that persist into adulthood (3, 4). Figure 1 depicts the range of health complications and impairments that present in children and adolescents with obesity and the related adult conditions where risk is increased with obesity during childhood (5–31).

There is evidence that interventions to improve nutrition, physical fitness and emotional health can lead to positive changes in behavior, diet, and physical activity. Multi-component interventions are proven more effective than single interventions at reducing body size and improving health-related quality of life in children and adolescents with obesity (32, 33). Multicomponent interventions are the first-line treatment for child and adolescent obesity with additional pharmacotherapy and surgical intervention required in more severe cases (3, 4).

Childhood obesity has increased in Ireland consistent with global trends, and Ireland now ranks 10^th^ in the EU for overweight and obesity in children aged 6–9 years (34). In little more than a generation, obesity among children in Ireland has increased 10-fold to affect around 9% of children in 2016 (2). 2018 trends suggest that the prevalence of childhood obesity in Ireland was plateauing (35) however, the prevalence for children living in disadvantage continued to rise. In turn, the leveling off of obesity prevalence previously seen at the population may be reversed due to the impact of the COVID-19 pandemic on child health and growth. For example, in England the findings of the National Child Measurement Programme (NCMP) for the 2020/2021 school year estimate that obesity prevalence increased from 9.9 to 14.4% and from 21 to 25.5% between 2019/20 and 2020/21 in children aged 4–5 years and 10–11 years respectively. Childhood obesity disproportionally affects low income families, and in Ireland, the prevalence of morbid obesity is 1.8% in the general population (7-year-old children) and 3.2% for those in disadvantage (36). Based on recent census this equates to approximately 21,000 children with severe obesity (37). Provision of clinical services to treat childhood obesity is important since multiple obesity-related health complications and comorbidities emerge in childhood (see Figure 1) such that children present to general pediatric and other pediatric specialities for evaluation and management (38). In addition, clinical services are required in line with Article 24 of the United Nations Convention on the Rights of the Child (access to healthcare). Treating childhood obesity is also important as obesity and related complications can track into adulthood, with ongoing consequences for the health and wellbeing of the individual, for future health services and for the Irish economy.

In Ireland, a Tier 3 (tertiary level) clinical service for the assessment and treatment of obesity in children and adolescents exists in Children's Health Ireland (CHI) at Temple Street The W28GO Child and Adolescent Obesity Service (www.w82go.ie) is a family-based, multi-disciplinary obesity service for individuals with obesity under 16 years of age. The service was developed based on international guidelines on treatment of childhood obesity (39, 40) and to date, process- and patient-level outcomes are positive (41). The service is a center of excellence accredited as a Pediatric Center for Obesity Management by the European Association for the Study of Obesity and has partial funding from the11 HSE/Department of Health with capacity to see approximately 120 new patients and 250 return patients each year. In 2021 a National Model of Care for the Management of Overweight and Obesity was launched in Ireland which highlighted the need to increase acces to childhood obesity services and to evaluate the health impacts of such interventions (42). To support integrated care and early intervention for children with obesity, it is essential to evaluate existing obesity interventions. Building an understanding of the health impacts of existing obesity interventions will support and enhance development and implementation of new interventions throughout primary, secondary and tertiary care. Preliminary data exist regarding the impacts of obesity treatment in pediatric healthcare in Ireland (41, 43–47) however it is unknown whether children from varied demographic or medical backgrounds have different outcomes from treatment for obesity.

This study aimed to explore the impact of the W82GO service on BMI-SDS and whether change in BMI-SDS differed between socioeconomic groups, sex, those with co-morbid conditions or the type of treatment offered.

## Materials and Methods

The participants of this study were all children and adolescents (<16 years) referred with a diagnosis of clinical obesity (BMI >98th percentile for age and sex) to the Tier 3 W82GO Child and Adolescent Weight Management Service at CHI at Temple Street, Dublin, Ireland. The service does not exclude children with disability or co-morbid diagnoses and as such any child referred by a hospital consultant (pediatric physician or surgeon) were included in the analysis. Audit approval (CA21-06-02) was granted by the hospital in order to explore routinely collected health data. The data for all children and adolescents presenting to the service between 2008 and 2019 were included in the study.

Following referral by pediatric physicians and surgeons, patients were assessed by a pediatric MDT (dietician, physiotherapist, psychologist and medical social worker) and a personalized treatment plan was developed and delivered as either individual care, group care or a combination of these (combined). Treatment involved delivery of a complex intervention incorporating nutrition therapy, exercise therapy, behavioral support and psychological/family therapy. Behavior change techniques where embedded in the intervention in line with clinical guidelines and the intervention was offered as a group programme or *via* individual outpatient appointments. Further description of the clinical service and intervention is available elsewhere (46) but briefly up to 24 clinical treatment sessions were offered to patients incorporating practical education related to nutrition (e.g., understanding food labeling and portion sizes, and learning how to shop in a supermarket), physical activity, fitness and function (e.g., improving fundamental motor skill and physical fitness, and supervised physical activity sessions) and behavior change (e.g., goal setting, self monitoring, stimulus control). In addition, education and advice addressed additional areas supporting behavior change (see Table 1). It was not possible to standardize the intervals between clinic visits as they are dependent on the multiple factors related to the individual (e.g., severity of obesity and related complications, distance from the clinic, parental availability and motivation, and treatment intensity recommended by the multidisciplinary team (MDT).

**Table 1:** Example components included in complex intervention for obesity treatment.

**Evidence-based intervention strategies and targets**
Education and information on the benefits of a healthy lifestyle (68–72)
Practical education and support for appropriate portion sizes (73)
Supporting reduced intake of sugar-sweetended drinks (74)
Facilitating attentive/mindful eating (75, 76)
Facilitating new cooking skills (77)
Supporting increased fruit and vegetable intake (78)
Supporting increased fiber intake (79)
Supporting reduced saturated fat intake (80, 81)
Supporting reduced frequency of take-away foods (82)
Supporting increased hydration (83)
Supporting swap of refined carbohydrates for those with a lower glycemic index (84)
Supporting removal of electronic distractions when eating and sleeping (85–87)
Supporting increased chewing of food and decrease rate of eating (88, 89)
Supporting increased sleep duration (90)
Supporting reduced time spent using television and screens (91)
Encouraging muting of television advertisements (92)
Facilitating practice of physical tasks and activities to increase self-efficacy (93)
Supporting increased levels of moderate-to-vigorous physical activity toward 60 min per day (94, 95)

Anthropometric (weight, height, body mass index (BMI) and BMI-SDS) on UK90 child growth charts (48), demographic information (sex and socioeconomic position [SEP] estimated with Pobal score, (data.gov.ie/dataset/pobal-hp-deprivation-index), and medical history were recorded at baseline. All anthropometric measurements and questionnaires related to physical activity, psychosocial health, sleep, quality of life and nutritional intake were administered by trained clinical staff. Body weight was measured using an electronic scale (SECA, Vogel & Halke, Hamburg, Germany) and height to the nearest 0.1 cm with a stadiometer (SECA, Vogel & Halke) in light clothing and without shoes. These measures were measured in triplicate and used to calculate BMI (weight kg/height m^2^), expressed as a standardized score (BMI-SDS) according to age- and sex-specific UK reference data (49). Children are classified as having obesity if they plot above the 98th centile for BMI on relevant sex and age adjusted growth charts (12), and as having morbid obesity if they plot above the 99.6th BMI centile on age and sex-adjusted charts. The presence of a diagnosed co-morbid condition was assessed by review of medical records and common co-morbid conditions included: epilepsy, psoriasis, asthma, dyspraxia, intellectual disability (ID), attention deficit hyperactivity disorder (ADHD), and autistic spectrum disorder (ASD).

Descriptive statistics were used to explore distribution, central tendency and variation in the demographic data collected during the baseline assessment of the patients. The change in BMI-SDS between baseline and the children's last visit to the clinic was assessed using a *t*-test, and multiple linear regression analysis was used to investigate the association of demographic factors on the change in BMI-SDS. The power of the regression model was 0.98 (assuming *P* = 0.05, *n* = 662, and predictor degrees of freedom = 12), which indicates that it is extremely likely to detect a change in SDS-BMI of 0.05 or more, should such an effect exist. Plots of residuals and fitted values were used to visually assess the assumptions of the multiple linear regression model that the regression errors were normally distributed, independent and with constant variance. Collinearity between the predictor variables was assessed using variance inflation factors, that did not exceed 1.5 for any of the predictors in the reported model. All analyses were performed using R version 3.6.2 and *P* < 0.05 was considered to represent statistical significance.

## Results

There were 1,097 children referred to the Tier 3 W82GO Service between 2008 and 2020. Of these, 856 (78%) attended an initial assessment appointment, and 692 (81% of attendees) returned for one or more follow-up appointments. There were 29 participants dropped from the regression model at this point due to missing values in one or more variables. The number of follow-up appointments ranged from 1 to 24. Most children (47%) attended individual appointments only, 42% attended a combination of group and individual appointments (combined), and 10% attended only group appointments. Most children remained in the W82GO Service for more than 1 year (median = 401, IQR = 474). There were 86 children 16 years or older on discharge from the clinic.

**Figure 2:**
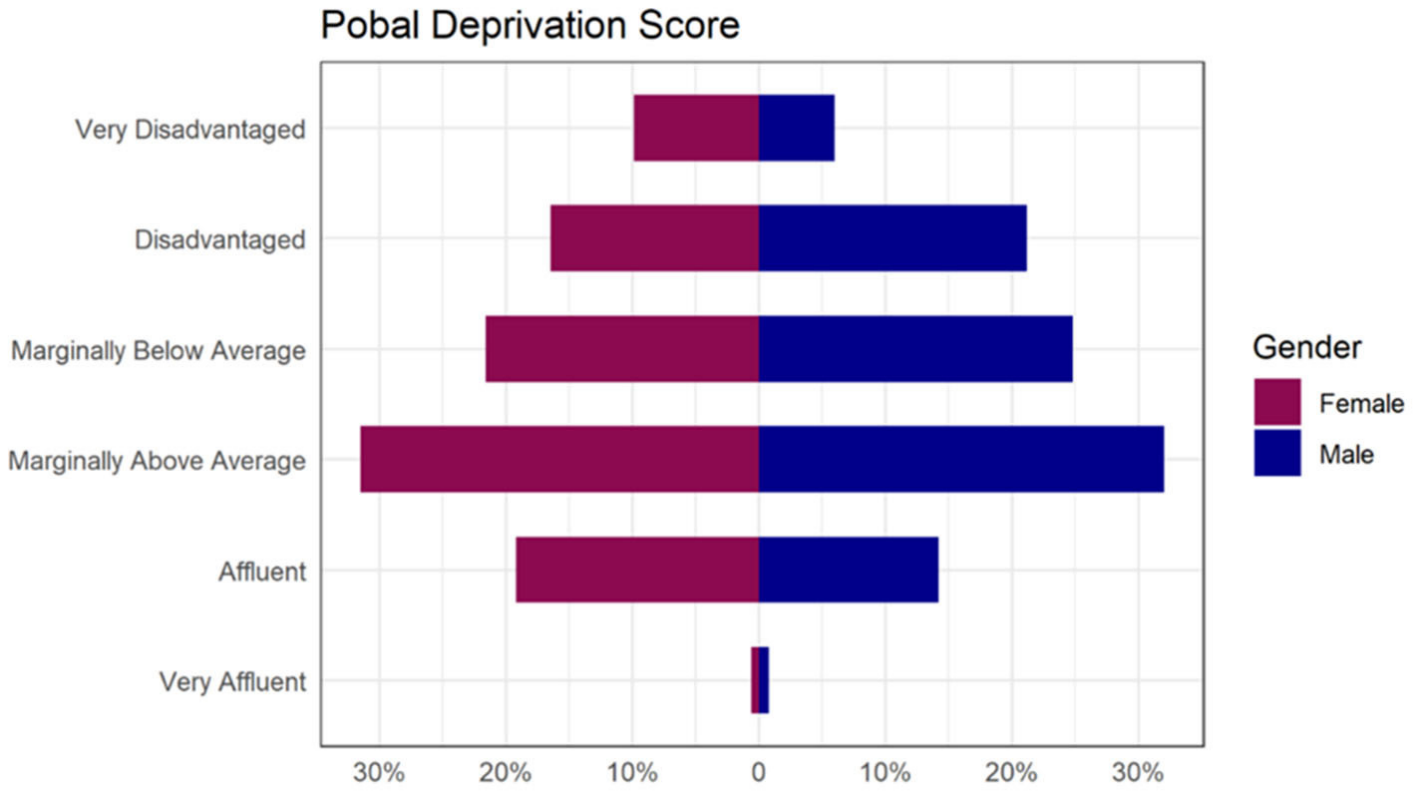
Proportion of children presenting to W82GO by Pobal Deprivation Score category illustrating that although the most common category was “Marginally above Average”, the data are strongly skewed toward disadvantage.

Mean age at baseline was 11 years, (SD = 6.5), 51% of children were female and 49% male. Most children (80%) had morbid obesity and the overall mean BMI-SDS was 3.17 (sd=0.62) at the baseline assessment appointment (Table 2). The Pobal deprivation index showed the distribution was skewed toward disadvantage), and 8% of the children were classed as very disadvantaged (Figure 2). In addition to obesity, 24% of children had one or more co-morbid conditions, including epilepsy, psoriasis, asthma, dyspraxia, ID, ADHD or ASD (Table 2).

**Table 2:** Demography and clinic presentation data for study participants.

	**Age < 6 years (*N* = 70)**	**Age 6–12 years (*N* = 349)**	**Age > 12 (*N* = 270)**	**Total (*N* = 692)**
**Gender**	
Female	40 (57 %)	175 (50 %)	142 (53 %)	359 (52 %)
Male	30 (43 %)	174 (50 %)	128 (47 %)	333 (48 %)
**Weight (kg)**	
Mean (SD)	31.13 (± 11.28)	59.40 (± 16.81)	93.62 (± 19.45)	69.90 (± 27.11)
**Height (cm)**	
Mean (SD)	112.5 (± 11.49)	143.3 (± 11.93)	164.6 (± 8.306)	148.4 (± 19.07)
**BMI (kg/m** ^**2**^ **)**	
Mean (SD)	24.07 (± 3.196)	28.23 (± 4.197)	34.36 (± 5.510)	30.18 (± 5.855)
**BMI SDS**	
Mean (SD)	3.749 (± 0.9853)	3.087 (± 0.5337)	3.130 (± 0.5226)	3.174 (± 0.6259)
**BMI percentile**	
Mean (SD)	99.79 (± 0.5330)	99.67 (± 0.9103)	99.70 (± 0.6447)	99.69 (± 0.7819)
**Pobal category**	
Very affluent	0 (0 %)	3 (1 %)	2 (1 %)	5 (1 %)
Affluent	15 (21 %)	58 (17 %)	42 (16 %)	115 (17 %)
Marginally above average	24 (34 %)	115 (33 %)	80 (30 %)	220 (32 %)
Marginally below Average	11 (16 %)	77 (22 %)	72 (27 %)	161 (23 %)
Disadvantaged	15 (21 %)	64 (18 %)	51 (19 %)	131 (19 %)
Very disadvantaged	4 (6 %)	28 (8 %)	23 (9 %)	55 (8 %)
**Body size category**	
Morbid obesity	62 (89 %)	274 (79 %)	219 (81 %)	556 (80 %)
Obesity	5 (7 %)	65 (19 %)	40 (15 %)	110 (16 %)
Overweight	3 (4 %)	6 (2 %)	6 (2 %)	15 (2 %)
**Treatment category**	
Individual	58 (83 %)	143 (41 %)	125 (46 %)	327 (47 %)
Mixed	12 (17 %)	173 (50 %)	108 (40 %)	294 (42 %)
Group	0 (0 %)	33 (9 %)	37 (14 %)	71 (10 %)
**BMI SDS change**	
Mean (SD)	−0.5150 (± 0.7863)	−0.1527 (± 0.4525)	−0.08938 (± 0.3283)	−0.1692 (± 0.4834)
**Comorbidities**	
Epilepsy	0 (0 %)	6 (2 %)	8 (3 %)	14 (2 %)
Asthma	5 (12 %)	39 (16 %)	37 (12 %)	81 (14 %)
Autism	0 (0 %)	10 (4 %)	3 (1 %)	13 (2 %)
ADHD	0 (0 %)	9 (4 %)	3 (1 %)	12 (2 %)
Intellectual disability	0 (0 %)	5 (2 %)	10 (3 %)	15 (3 %)
Dyspraxia	0 (0 %)	7 (3 %)	7 (2 %)	14 (2 %)
Psoriasis	0 (0 %)	1 (0 %)	4 (1 %)	5 (1 %)

**Figure 3:**
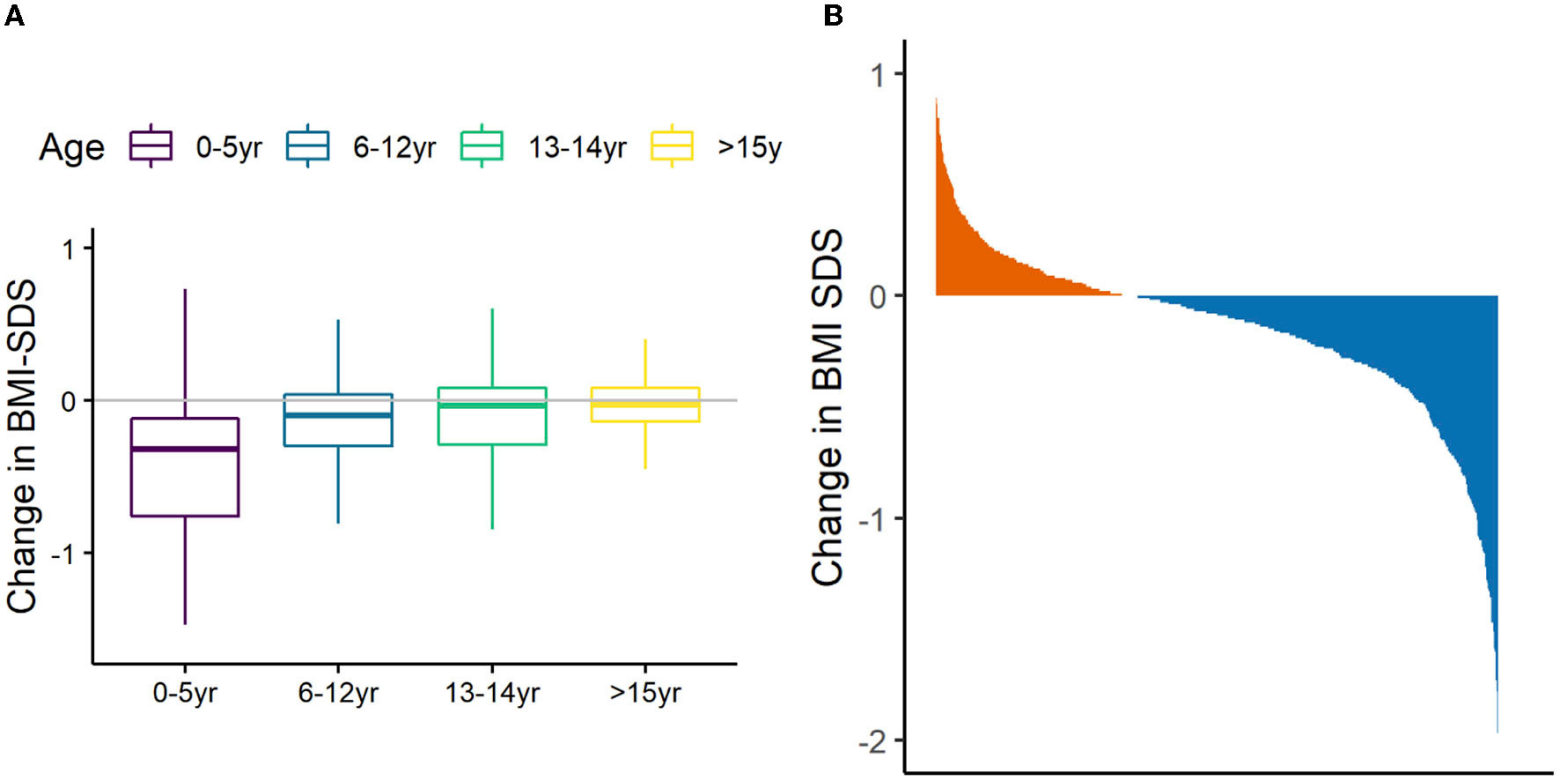
Relationship between age and change in BMI-SDS the 692 children referred to W82GO. Data are mean (bar), interquartile range (box), and 1.5*IQR (whiskers) **(A)**. Waterfall plot showing overall proportion of children that had lost (blue) weight on discharge from the W82GO clinic **(B)**.

The mean BMI-SDS change across the whole cohort on discharge was −0.17 (95% CI = −0.20, −0.13; *P* < 0.001). Most children (64%) had lower BMI SDS after treatment, 34% gained and 2% had no change (Figure 3). Younger age at admission was associated with greater reduction in BMI SDS (β = 0.03, 95% CI = 0.02, 0.04, *P* < 0.001). Table 3 describes regression analysis where longer treatment duration was associated with greater reduction of BMI SDS (β = −0.01; 95% CI = −0.14, −0.06; *P* < 0.001). There was no significant association between reduction in BMI SDS and any of the other parameters (Pobal score, treatment type, sex, comorbid condition).

**Table 3:** Multiple linear regression analysis of the association between change in SDS-BMI and demographic parameters.

**Predictors**	**Change in BMI-SDS**		
	**Estimates**	**CI**	***P*-Value**
**Age**	0.03	0.02 to 0.04	**<0.001**
**Body size category** ref = Morbid Obesity
Obesity	0.06	−0.03 to 0.16	0.199
Overweight	0.07	−0.17 to 0.31	0.564
**Gender** Ref = Female
Male	−0.01	−0.08 to 0.06	0.827
**Treatment category** ref = Group
Individual	0.03	−0.09 to 0.15	0.613
Mixed	0.10	−0.03 to 0.22	0.131
**Pobal category** Ref = very affluent
Affluent	0.18	−0.23 to 0.60	0.394
Marginally above average	0.21	−0.20 to 0.62	0.314
Marginally below average	0.19	−0.22 to 0.60	0.370
Disadvantaged	0.23	−0.19 to 0.64	0.291
Very disadvantaged	0.19	−0.23 to 0.62	0.380
**Treatment duration (years)**	−0.10	−0.14 to −0.06	**<0.001**
**Comorbidity**	−0.02	−0.10 to 0.07	0.689
Observations	663
*R* ^2^	0.135

*Bold values are statistically significant predictors*.

## Discussion

The results of this study demonstrate that engagement in a Tier 3 specialist pediatric obesity service was associated with a significant reduction in BMI-SDS in children with obesity in Ireland. Reduction in BMI-SDS was greater in younger children, and in children with longer treatment durations, but was not affected by sex, treatment type (group/individual), obesity category on admission, co-morbid conditions, or SEP. In agreement with other reports of multidisciplinary childhood obesity interventions, there was extensive between-subject variability in the response to treatment, and in the parameters recorded at baseline.

Most children reduced BMI-SDS when engaged with the W82GO Service, but the absolute changes in BMI-SDS were moderate (mean BMI-SDS reduction = 0.17). These data were collected in a clinical service setting, and the findings are directly applicable to the clinical cohort and treatment challenges of childhood obesity in the Irish health service. The BMI-SDS reduction we report for the W82GO service is comparable to other European multi-disciplinary interventions. For example, the changes in BMI-SDS are similar to those reported in a 2018 systematic review of Tier 3 pediatric weight management interventions from the UK (50), and to a shorter term (10-week) community-based intervention in the UK (MEND) which reported a mean 0.18-unit reduction in BMI-SDS when applied in 9,563 children (51). Furthermore, systematic reviews of randomized controlled trials of multidisciplinary interventions in childhood obesity reported a mean reduction in BMI-SDS of 0.13 units in 2,399 participants of 20 trials (32), and a reduction of 0.20 in 12 trials (50). Therefore, our findings of moderate reduction in BMI-SDS in most patients over the 12 years of implementing W82GO are consistent with the findings from similar interventions reported elsewhere.

There was a significant association between age at admission and reduced BMI SDS in this study, suggesting that children referred to the W82GO Service at a younger age had greater improvements in BMI-SDS. Previous studies have reported inconsistent associations between the success of multidisciplinary interventions in obesity and age at admission (51–53). This may reflect differences between children and adolescents including their stage of development, the length of time exposed to the state of obesity (which makes treatment more intractable) or the differing treatment approaches used for children at different stages of development. Parents may have more success at modifying the home environment of younger children, compared to adolescents where social and peer influence may be stronger. In agreement with other studies of multidisciplinary obesity interventions (52, 53), there was no association between sex and BMI-SDS reduction in our study.

There is extensive evidence that obesity is associated with SEP with higher prevalence typically reported in children in disadvantaged categories (54). Recent population-level data in the UK from 1.2 million children of comparable age, sex and ethnicity to W82GO clearly shows a strong and linear relationship between obesity and SEP (54). In contrast, “marginally above average” was the most common Pobal deprivation category of the children in this study, suggesting that those attending W82GO do not represent the children in Ireland with the highest risk of obesity, and that families living with severe deprivation may either not be referred as often to the service or may fail to attend the service. As such, considerations regarding how the service engages and supports families living in disadvantage is warranted. There are no stratified data on the prevalence of obesity by SEP in Irish children, (55, 56) but previous studies have reported increased prevalence of obesity in schools in disadvantaged areas in Ireland (57). Children from lower SEP are more likely to drop out of obesity interventions (51) and less likely to sustain reductions in obesity once treatment has ended (58). However, it is unknown as to whether they are more or less likely to be referred for treatment in the first instance. The strong relationship between SEP and childhood obesity should be a primary consideration for all interventions targeted at prevention and treatment of childhood obesity Development of Irish childhood obesity services should specifically target recruitment and retention of children in lower SEP categories to address inequality and the greater burden of risk. In addition, social and community-based integrated care will be essential for such high risk and vulnerable groups.

There are many reports describing the efficacy of multidisciplinary childhood obesity interventions, but comparisons between studies are precluded by the diverse range of recruitment/referral processes, clinical settings, training of clinical staff, demography, focus and delivery of therapies, and timing and duration of treatment. Perhaps the only consistency across these studies is the use of BMI-SDS to monitor response to treatment. BMI-SDS is a simple, quantitative measure of body size that facilitates comparison across growth intervals, yet despite its almost universal application, BMI-SDS poorly represents the metabolic, physical, and psychological pathology of obesity. In terms of quantification of body size, BMI-SDS is not an optimal predictor of adiposity in children with severe obesity, which defines a large proportion of children treated at obesity clinics (59). Furthermore, there is no consensus on the change in BMI-SDS that is associated with clinically significant changes in body fat or health outcomes at a population level. A recent meta-analysis proposed that a reduction in BMI-SDS of more than 0.6 units is required to ensure reduced body fat (60), and > 1.0, >1.2, and >0.7 for meaningful reductions in systolic blood pressure, low density lipoproteins and triglycerides, respectively (61). Despite these significant limitations, BMI-SDS is a low-cost, quick and easy to use metric employed to assess the efficacy of childhood obesity interventions (62). Future research should be directed toward development of holistic parameters that can assess the impact of obesity on child health and wellbeing and that can be used to reliably define and monitor ‘success’ in the evaluation of treatment interventions. We suggest that BMI-SDS be supplemented with measures of nutritional status, physical fitness, mental health, and musculoskeletal function rather than BMI SDS alone. Such additional parameters target the functional limitations that obesity imposes on children, and quantify improvements in quality of life that are not represented by the commonly used metrics of BMI, weight or BMI-SDS. Though our service integrates such measures as part of routine care, this paper did not focus on the outcomes related to quality of life, fitness, mental health or function and it is warranted to include this in future studies undertaken with the service.

Our findings must be interpreted in the context of numerous limitations. It was not possible to standardize treatment schedules between individuals, nor to compare patients to an untreated control group which could be considered unethical (63, 64). Consequently, the data are observational, and the possibility that changes in BMI-SDS were affected by factors other than the W82GO Service cannot be excluded. Nevertheless, the changes in BMI-SDS that we report are comparable to those reported in previous studies of multi-disciplinary childhood obesity interventions (33, 50, 60). It is a limitation of this study that it was not possible to standardize the timing or reasons for discharge from the clinic. There was no long-term (>24 months) follow-up in this analysis, and it is not known if the observed changed in BMI-SDS treatment were maintained once patients left the service.

This study tracks the progress of 692 children through the W82GO Service of Children's Health Ireland at Temple Street, and this large sample size and the longitudinal design are strengths. It is also a strength of this study that none of the parameters in this study were self-reported, all measurements were collected by clinical staff and indexed to UK and Ireland reference parameters for SEP and growth. This retrospective analysis of 12 years of multidisciplinary intervention for childhood obesity demonstrated moderate reductions in BMI-SDS in most children. There was extensive between-subject variability in the response to treatment, and in the parameters recorded at baseline, reflecting the multi-dimensional pathophysiology of childhood obesity, and supporting the W82GO approach of personalized intervention. Future research should focus on exploring whether children engaged in the service experienced change in other health outcomes including blood pressure, physical fitness, emotional- or mental-health outcomes or quality of life. In addition, further investigation is required to understand why some children do not respond to the intervention and how future interventions can optimally manage diverse phenotypes of obesity.

The Irish government commitments to address obesity include the development and implementation of a national integrated service model for the health and social care of those with obesity “including specialist services on an equitable geographic basis for both children and adults” (65). Multicomponent clinical services such as the W28GO Service are recommended in the RCPI Expert Report addressing obesity treatment (66) and further development of the W28GO Service is planned as part of the Model of Care for the Management of Overweight and Obesity developed by the National Clinical Programme for Obesity (67).

Clinical services such as W82GO and research studies indicate that childhood obesity has a multifactorial etiology that is driven by genetic, social, pathological and lifestyle-related factors. Future childhood obesity services should reflect this complexity by incorporating multidisciplinary interventions and considering systemic clinical outcomes in addition to changes in BMI-SDS.

## Data Availability Statement

The original contributions presented in the study are included in the article/Supplementary Materials, further inquiries can be directed to the corresponding author.

## Ethics Statement

The study was reviewed and approved by the Clinical Audit Committee of Children's Health Ireland at Temple Street. Written informed consent from the participant's legal guardian/next of kin was not required due to the audit of routinely collected and anonymised clinical data.

## Author Contributions

GO'M led the development of the clinical intervention, study design, and managed and directed the study. All authors made significant contributions to data collection. Data analysis was completed by GO'M and CW. All authors contributed to writing and approved the final manuscript.

## Funding

The work described in this study was funded by the Temple Street Foundation, the Health Research Board of Ireland and the RCSI Strategic Academic Recruitment (StAR) Fellowship.

## Conflict of Interest

The authors declare that the research was conducted in the absence of any commercial or financial relationships that could be construed as a potential conflict of interest.

## Publisher's Note

All claims expressed in this article are solely those of the authors and do not necessarily represent those of their affiliated organizations, or those of the publisher, the editors and the reviewers. Any product that may be evaluated in this article, or claim that may be made by its manufacturer, is not guaranteed or endorsed by the publisher.

## References

[1] Johnson W Li L Kuh D Hardy R. How has the age-related process of overweight or obesity development changed over time? Co-ordinated analyses of individual participant data from five United Kingdom Birth Cohorts. PLoS Med. (2015) 12:e1001828. 10.1371/journal.pmed.1001828 25993005 PMC4437909

[2] Bentham J Di Cesare M BIlano V Boddy L. Worldwide trends in children's and adolescents' body mass index, underweight and obesity, in comparison with adults, from 1975 to 2016: a pooled analysis of 2,416 population-based measurement studies with 128.9 million participants. Lancet. (2017). 390:2627–42. 10.1016/S0140-6736(17)32129-3 29029897 PMC5735219

[3] Jebeile H Kelly AS O'Malley G Baur LA. Obesity in children and adolescents: epidemiology, causes, assessment, and management. Lancet Diabetes Endocrinol. (2022). 10.1016/S2213-8587(22)00047-X 35248172 PMC9831747

[4] O'Malley GC Shultz SP Thivel D Tsiros MD. Neuromusculoskeletal health in pediatric obesity: incorporating evidence into clinical examination. Curr Obes Rep. (2021) 10:467–77. 10.1007/s13679-021-00463-9 34958437 PMC8727388

[5] Brara SM Koebnick C Porter AH Langer-Gould A. Pediatric idiopathic intracranial hypertension and extreme childhood obesity. J Pediatr. (2012) 161:602–7. 10.1016/j.jpeds.2012.03.047 22633290 PMC3572898

[6] Thang Le VN Kim J-G Yang Y-M Lee D-WJPD. Risk factors for early childhood caries: an umbrella review. Pediatr Dent. (2021) 43:176–94. 34172110

[7] Friedemann C Heneghan C Mahtani K Thompson M Perera R Ward AM. Cardiovascular disease risk in healthy children and its association with body mass index: systematic review and meta-analysis. BMJ. (2012) 345:e4759. 10.1136/bmj.e4759 23015032 PMC3458230

[8] Meyer AA Kundt G Steiner M Schuff-Werner P Kienast W. Impaired flow-mediated vasodilation, carotid artery intima-media thickening, and elevated endothelial plasma markers in obese children: the impact of cardiovascular risk factors. Pediatrics. (2006) 117:1560. 10.1542/peds.2005-2140 16651309

[9] Hudson LD Rapala A Khan T Williams B Viner RM. Evidence for contemporary arterial stiffening in obese children and adolescents using pulse wave velocity: a systematic review and meta-analysis. Atherosclerosis. (2015) 241:376–86. 10.1016/j.atherosclerosis.2015.05.014 26071661

[10] Pulgarón ER. Childhood obesity: a review of increased risk for physical and psychological comorbidities. Clin Ther. (2013) 35:A18–32. 10.1016/j.clinthera.2012.12.014 23328273 PMC3645868

[11] Jebeile H Lister NB Baur LA Garnett SP Paxton SJ. Eating disorder risk in adolescents with obesity. Obes Rev. (2021) 22:e13173. 10.1111/obr.13173 33410207

[12] Jebeile H Cardel MI Kyle TK Jastreboff AM. Addressing psychosocial health in the treatment and care of adolescents with obesity. Obesity. (2021) 29:1413–22. 10.1002/oby.23194 34431234

[13] Quek YH Tam WWS Zhang MWB Ho RCM. Exploring the association between childhood and adolescent obesity and depression: a meta-analysis. Obes Rev. (2017) 18:742–54. 10.1111/obr.12535 28401646

[14] Griffiths LJ Parsons TJ Hill AJ. Self-esteem and quality of life in obese children and adolescents: a systematic review. Int J Pediatr Obes. (2010) 5:282–304. 10.3109/17477160903473697 20210677

[15] Spilsbury JC Storfer-Isser A Rosen CL Redline SJS. Remission and incidence of obstructive sleep apnea from middle childhood to late adolescence. Sleep. (2015) 38:23–9. 10.5665/sleep.4318 25325456 PMC4262952

[16] Deng X Ma J Yuan Y Zhang Z Niu W. Association between overweight or obesity and the risk for childhood asthma and wheeze: an updated meta-analysis on 18 articles and 73 252 children. Pediatr Obes. (2019) 14:e12532. 10.1111/ijpo.12532 31033249

[17] Díez-Fernández A Sánchez-López M Mora-Rodríguez R Notario-Pacheco B Torrijos-Niño C Martínez-Vizcaíno V. Obesity as a mediator of the influence of cardiorespiratory fitness on cardiometabolic risk: a mediation analysis. Diabetes Care. (2014) 37:855–62. 10.2337/dc13-0416 24198304

[18] Thivel D Ring-Dimitriou S Weghuber D Frelut M-L O'Malley G. Muscle strength and fitness in pediatric obesity: a systematic review from the european childhood obesity group. Obes Facts. (2016) 9:52–63. 10.1159/000443687 26901423 PMC5644904

[19] Redline S Tishler PV Schluchter M Aylor J Clark K Graham GJAjor. Risk factors for sleep-disordered breathing in children: associations with obesity, race, and respiratory problems *Am J Respir Crit Care Med*. (1999) 159:1527–32. 10.1164/ajrccm.159.5.9809079 10228121

[20] Nussbaum BM Mathew MS Atem F Barlow SE Gupta OT Messiah SE. Distribution of comorbidities as primary diagnoses by obesity class among patients in a large us paediatric healthcare system. Clin Obes. (2021) 11:e12478. 10.1111/cob.12478 34250735

[21] Reinehr T Roth CL. Is there a causal relationship between obesity and puberty? Lancet Child Adolesc Health. (2019) 3:44–54. 10.1016/S2352-4642(18)30306-7 30446301

[22] Li L Feng Q Ye M He Y Yao A Shi K. Metabolic effect of obesity on polycystic ovary syndrome in adolescents: a meta-analysis. J Obstet Gynaecol. (2017) 37:1036–47. 10.1080/01443615.2017.1318840 28657375

[23] Tsiros MD Tian EJ Shultz SP Olds T Hills AP Duff J. Obesity, the new childhood disability? An umbrella review on the association between adiposity and physical function. Obes Rev. (2020) 21:e13121. 10.1111/obr.13121 32779327

[24] Molina-Garcia P Migueles JH Cadenas-Sanchez C Esteban-Cornejo I Mora-Gonzalez J Rodriguez-Ayllon M. A systematic review on biomechanical characteristics of walking in children and adolescents with overweight/obesity: possible implications for the development of musculoskeletal disorders. Obes Rev. (2019) 20:1033–44. 10.1111/obr.12848 30942558

[25] Chan G Chen CT. Musculoskeletal effects of obesity. Curr Opin Pediatr. (2009) 21:65–70. 10.1097/MOP.0b013e328320a914 19242242

[26] Adelman RD Restaino IG Alon US. Blowey DL. Proteinuria and focal segmental glomerulosclerosis in severely obese adolescents. J Pediatr. (2001) 138:481–5. 10.1067/mpd.2001.113006 11295709

[27] Haid B Tekgül S. Primary and secondary enuresis: pathophysiology, diagnosis, and treatment. Eur Urol Focus. (2017) 3:198–206. 10.1016/j.euf.2017.08.010 28888814

[28] Eslam M Alkhouri N Vajro P Baumann U Weiss R Socha P. Defining paediatric metabolic (dysfunction)-associated fatty liver disease: an international expert consensus statement. Lancet Gastroenterol Hepatol. (2021) 6:864–73. 10.1016/S2468-1253(21)00183-7 34364544

[29] Maguolo A Maffeis C. Acanthosis nigricans in childhood: a cutaneous marker that should not be underestimated, especially in obese children. Acta Paediatr. (2020) 109:481–7. 10.1111/apa.15031 31560795

[30] Thivel D Isacco L O'Malley G Duché P. Pediatric obesity and perceived exertion: difference between weight-bearing and non-weight-bearing exercises performed at different intensities. J Sports Sci. (2016) 34:389–94. 10.1080/02640414.2015.1061200 26090822

[31] Tompkins CL Flanagan T Lavoie J Brock DW. Heart rate and perceived exertion in healthy weight and obese children during a self-selected physical activity program. J Phys Act Health. (2015) 12:976–81. 10.1123/jpah.2013-0374 25203162

[32] Mead E Brown T Rees K Azevedo LB Whittaker V Jones D. Diet, Physical activity and behavioural interventions for the treatment of overweight or obese children from the age of 6 to 11 years. Cochrane Database Syst Rev. (2017) 6:CD012651. 10.1002/14651858.CD012651 28639319 PMC6481885

[33] Ells LJ Rees K Brown T Mead E Al-Khudairy L Azevedo L. Interventions for treating children and adolescents with overweight and obesity: an overview of cochrane reviews. Int J Obes. (2018) 42:1823–33. 10.1038/s41366-018-0230-y 30301964

[34] Spinelli A Buoncristiano M Kovacs VA Yngve A Spiroski I Obreja G. Prevalence of severe obesity among primary school children in 21 European Countries. Obes Facts. (2019) 12:244–58. 10.1159/000500436 31030201 PMC6547273

[35] O'Donnell A Buffini M Kehoe L Nugent A Kearney J Walton J. The prevalence of overweight and obesity in irish children between 1990 and 2019. Public Health Nutr. (2020) 23:2512–20. 10.1017/S1368980020000920 32613932 PMC10200483

[36] Bel Serrat S HM O'Malley G Mehegan J Murrin C Kelleher CK. Trends in the prevalence of childhood obesity and morbid obesity in the Republic of Ireland – the childhood obesity surveillance initiative in 2008, 2010, 2012 and 2015. Obes Facts. (2018) 11:43. 10.1159/000497797

[37] Heinen MM Murrin C Daly L O'Brien J Heavey P Kilroe J. The Childhood Obesity Surveillance Initiative (COSI) in the Republic of Ireland: Findings from 2008, 2010 2012. (2008). Available online at: http://www.ucd.ie/t4cms/COSI%20report

[38] Tully L Sorensen J O'Malley G. Hospital service use among children with obesity in ireland: a micro-costing study. Child Care Pract. (2022) 1−17. 10.1080/13575279.2022.2035682

[39] Logue J Thompson L Romanes F Wilson DC Thompson J Sattar NJB. management of obesity: summary of sign guideline. BMJ. (2010) 340:c154. 10.1136/bmj.c154 20181637

[40] NICE CfPHEa Care NCCfP. Obesity: The Prevention, Identification, Assessment and Management of Overweight and Obesity in Adults and Children [Internet]. London: National Institute for Health and Clinical Excellence (2006). 22497033

[41] O'Malley G Brinkley A Moroney K McInerney M Butler J Murphy N. Is the temple street W82go healthy lifestyles programme effective in reducing Bmi Sds? 833 Accepted Poster. Obesity Facts. (2012) 5:223. 10.1159/000258190

[42] Obesity NCPf. Mode of Care for the Management of Overweight and Obesity. Dublin: Health Service Executive (2021).

[43] Browne S Kechadi M-T O'Donnell S Dow M Tully L Doyle G. Mobile health apps in pediatric obesity treatment: process outcomes from a feasibility study of a multicomponent intervention. JMIR mHealth uHealth. (2020) 8:e16925. 10.2196/16925 32673267 PMC7381070

[44] Kelleher E Davoren MP Harrington JM Shiely F Perry IJ McHugh SM. Barriers and facilitators to initial and continued attendance at community-based lifestyle programmes among families of overweight and obese children: a systematic review. Obes Rev. (2017) 18:183–94. 10.1111/obr.12478 27862851 PMC5245104

[45] Kelleher E Harrington JM Shiely F Perry IJ McHugh SM. Barriers and facilitators to the implementation of a community-based, multidisciplinary, family-focused childhood weight management programme in Ireland: a qualitative study. BMJ Open. (2017) 7:e016459. 10.1136/bmjopen-2017-016459 28851786 PMC5623413

[46] O'Malley GC. Childhood Obesity Treatment: Integrating Mobile Health Technology into a Paediatric Obesity Service. Cork: University College Cork (2015).

[47] Tully L Sorensen J O'Malley G. Pediatric weight management through mhealth compared to face-to-face care: cost analysis of a randomized control trial. JMIR mHealth and uHealth. (2021) 9:e31621. 10.2196/31621 34519665 PMC8479601

[48] Cole T. Growth monitoring with the British 1990 growth reference. Arch Dis Child. (1997) 76:47–9. 10.1136/adc.76.1.47 9059161 PMC1717048

[49] Cole TJ. Using the Lms method to measure skewness in the Nchs and Dutch National Height Standards. Ann Hum Biol. (1989) 16:407–19. 10.1080/03014468900000532 2802520

[50] Brown T O'Malley C Blackshaw J Coulton V Tedstone A Summerbell C. Exploring the evidence base for tier 3 specialist weight management interventions for children aged 2–18 years in the Uk: a rapid systematic review. J Public Health. (2017) 40:835–47. 10.1093/pubmed/fdx166 29228233

[51] Fagg J Chadwick P Cole TJ Cummins S Goldstein H Lewis H. From trial to population: a study of a family-based community intervention for childhood overweight implemented at scale. Int J Obes. (2014) 38:1343–9. 10.1038/ijo.2014.103 24919564 PMC4175967

[52] Reinehr T Temmesfeld M Kersting M De Sousa G Toschke AM. Four-year follow-up of children and adolescents participating in an obesity intervention program. Int J Obes. (2007) 31:1074–7. 10.1038/sj.ijo.0803637 17471300

[53] Mameli C Krakauer JC Krakauer NY Bosetti A Ferrari CM Schneider L. Effects of a multidisciplinary weight loss intervention in overweight and obese children and adolescents: 11 years of experience. PLoS ONE. (2017) 12:e0181095. 10.1371/journal.pone.0181095 28704494 PMC5509286

[54] Strugnell C Mathrani S Sollars L Swinburn B Copley V. Variation in the socioeconomic gradient of obesity by ethnicity–England's National Child Measurement Programme. Obesity. (2020) 28:1951–63. 10.1002/oby.22970 32886431 PMC7540500

[55] Madden D. Bmi mobility and obesity transitions among children in Ireland. Econ Hum Biol. (2020) 38:100896. 10.1016/j.ehb.2020.100896 32526642

[56] Madden D. Child and Adolescent Obesity in Ireland: A longitudinal perspective. Dublin: UCD Centre for Economic Research Working Paper Series (2016).

[57] Bel-Serrat S Heinen MM Mehegan J O'Brien S Eldin N Murrin CM. School sociodemographic characteristics and obesity in schoolchildren: does the obesity definition matter? BMC Public Health. (2018) 18:1–12. 10.1186/s12889-018-5246-7 29523113 PMC5845160

[58] Plachta-Danielzik S Landsberg B Lange D Seiberl J Müller MJ. Eight-year follow-up of school-based intervention on childhood overweight–the kiel obesity prevention study. Obes Facts. (2011) 4:35–43. 10.1159/000324552 21372609 PMC6444753

[59] Freedman DS Butte NF Taveras EM Lundeen EA Blanck HM Goodman AB. Bmi Z-scores are a poor indicator of adiposity among 2-to 19-year-olds with very high Bmis, Nhanes 1999-2000 to 2013-2014. Obesity. (2017) 25:739–46. 10.1002/oby.21782 28245098 PMC5373980

[60] Birch L Perry R Hunt LP Matson R Chong A Beynon R. What change in body mass index is associated with improvement in percentage body fat in childhood obesity? A meta-regression. BMJ Open. (2019) 9:e028231. 10.1136/bmjopen-2018-028231 31473614 PMC6720247

[61] El-Medany AYM Birch L Hunt LP Matson RIB Chong AHW Beynon R. What Change in body mass index is required to improve cardiovascular outcomes in childhood and adolescent obesity through lifestyle interventions: a meta-regression. Childhood Obesity. (2020) 16:449–78. 10.1089/chi.2019.0286 32780648 PMC7575353

[62] Savoye M Nowicka P Shaw M Yu S Dziura J Chavent G. Long-term results of an obesity program in an ethnically diverse pediatric population. Pediatrics. (2011) 127:402–10. 10.1542/peds.2010-0697 21300674 PMC3065145

[63] Leproult R Holmbäck U Van Cauter E. Circadian misalignment augments markers of insulin resistance and inflammation, independently of sleep loss. Diabetes. (2014) 63:1860–9. 10.2337/db13-1546 24458353 PMC4030107

[64] Holm JC Nowicka P Farpour-Lambert NJ O'Malley G Hassapidou M Weiss R. The ethics of childhood obesity treatment - from the Childhood Obesity Task Force (Cotf) of European Association for the Study of Obesity (Easo). Obes Facts. (2014) 7:274–81. 10.1159/000365773 25096302 PMC5644819

[65] Department of Health. A Healthy Weight for Ireland: Obesity Policy and Action Plan 2016-2025. Dublin: The Stationery Office (2016).

[66] Obesity RPGo. An Expert Report on How to Clinically Manage and Treat Obesity in Ireland. Dublin (2015).

[67] Obesity NCPf. Model of Care for the Management of Overweight and Obesity. Dublin: RCPI (2021).

[68] Bandura A. Health promotion by social cognitive means. Health Educ Behav. (2004) 31:143–64. 10.1177/1090198104263660 15090118

[69] Janz NK Champion VL Strecher VJ. The health belief model. In: Glanz K, Rimer BK, Lewis FM, editor. Health Behavior and Health Education: Theory, Research Adn Practice, 3rd ed. San Francisco, CA: Jossey-Bass (2002), p. 65.

[70] Prochaska JO Velicer WF. The transtheoretical model of health behavior change. Am J Health Promot. (1997) 12:38–48. 10.4278/0890-1171-12.1.38 10170434

[71] Ajzen I. The theory of planned behavior. Organ Behav Hum Decis Process. (1991) 50:179–211. 10.1016/0749-5978(91)90020-T

[72] Goldfield GS Henderson K Buchholz A Obeid N Nguyen H Flament MF. Physical activity and psychological adjustment in adolescents. J Phys Act Health. (2011) 8:157–63. 10.1123/jpah.8.2.157 21415442

[73] Swinburn B Sacks G Ravussin E. Increased food energy supply is more than sufficient to explain the US epidemic of obesity. Am J Clin Nutr. (2009) 90:1453–6. 10.3945/ajcn.2009.28595 19828708

[74] Ludwig DS Peterson KE Gortmaker SL. Relation between consumption of sugar-sweetened drinks and childhood obesity: a prospective, observational analysis. Lancet. (2001) 357:505–8. 10.1016/S0140-6736(00)04041-1 11229668

[75] Higgs S Williamson A Rothstein P Humphreys G. Sensory specific satiety is intact in amnesics who eat multiple meals. Psychol Sci. (2008) 19:623–8. 10.1111/j.1467-9280.2008.02132.x 18727773

[76] Rozin P Dow S Moscovitch M Rajaram S. What causes humans to begin and end a meal? A role for memory for what has been eaten, as evidence by a study of multiple meal eating in amnesic patients. Psychol Sci. (1998) 9:392–6. 10.1111/1467-9280.00073

[77] Reicks M Trofholz AC Stang JS Laska MN. Impact of cooking and home food preparation interventions among adults: outcomes and implications for future programs. J Nutr Educ Behav. (2014) 46:259–76. 10.1016/j.jneb.2014.02.001 24703245 PMC4063875

[78] Rockett HR Berkey CS Field AE Colditz GA. Cross-sectional measurement of nutrient intake among adolescents in 1996. Prev Med. (2001) 33:27–37. 10.1006/pmed.2001.0850 11482993

[79] Johnson L Mander AP Jones LR Emmett PM Jebb SA. Energy-dense, low-fiber, high-fat dietary pattern is associated with increased fatness in childhood. Am J Clin Nutr. (2008) 87:846–54. 10.1093/ajcn/87.4.846 18400706

[80] Jequier E. Is fat intake a risk factor for fat gain in children? J Clin Endocrinol Metab. (2001) 86:980–3. 10.1210/jc.86.3.980 11238472

[81] Kris-Etherton P Daniels SR Eckel RH Engler M Howard BV Krauss RM. Summary of the scientific conference on dietary fatty acids and cardiovascular health: conference summary from the nutrition committee of the American Heart Association. Circulation. (2001) 103:1034–9. 10.1161/01.CIR.103.7.1034 11181482

[82] Bowman SA Gortmaker SL Ebbeling CB Pereira MA Ludwig DS. Effects of fast-food consumption on energy intake and diet quality among children in a national household survey. Pediatrics. (2004) 113:112–8. 10.1542/peds.113.1.112 14702458

[83] Stahl A Kroke A Bolzenius K Manz F. Relation between hydration status in children and their dietary profile - results from the donald study. Eur J Clin Nutr. (2007) 61:1386–92. 10.1038/sj.ejcn.1602663 17311062

[84] Toeller M Buyken AE Heitkamp G Cathelineau G Ferriss B Michel G. Nutrient intakes as predictors of body weight in European people with type 1 diabetes. Int J Obes Relat Metab Disord. (2001) 25:1815–22. 10.1038/sj.ijo.0801816 11781763

[85] Foley LS Maddison R Jiang Y Marsh S Olds T Ridley K. Presleep activities and time of sleep onset in children. Pediatrics. (2013) 131:276–82. 10.1542/peds.2012-1651 23319532

[86] Arora T Hussain S Hubert Lam KB Lily Yao G Neil Thomas G Taheri S. Exploring the complex pathways among specific types of technology, self-reported sleep duration and body mass index in UK adolescents. Int J Obes. (2013) 37:1254–60. 10.1038/ijo.2012.209 23295500

[87] Bickham DS Blood EA Walls CE Shrier LA Rich M. Characteristics of screen media use associated with higher Bmi in young adolescents. Pediatrics. (2013) 131:935–41. 10.1542/peds.2012-1197 23569098 PMC3639454

[88] Higgs S Jones A. Prolonged chewing at lunch decreases later snack intake. Appetite. (2013) 62:91–5. 10.1016/j.appet.2012.11.019 23207188

[89] Karl JP Young AJ Rood JC Montain SJ. Independent and combined effects of eating rate and energy density on energy intake, appetite, and gut hormones. Obesity. (2013) 21:E244–52. 10.1002/oby.20075 23592679

[90] Iglowstein I Jenni OG Molinari L Largo RH. Sleep duration from infancy to adolescence: reference values and generational trends. Pediatrics. (2003) 111:302–7. 10.1542/peds.111.2.302 12563055

[91] Wiecha JL Peterson KE Ludwig DS Kim J Sobol A Gortmaker SL. When children eat what they watch: impact of television viewing on dietary intake in youth. Arch Pediatr Adolesc Med. (2006) 160:436–42. 10.1001/archpedi.160.4.436 16585491

[92] Halford JC Boyland EJ Hughes GM Stacey L McKean S Dovey TM. Beyond-brand effect of television food advertisements on food choice in children: the effects of weight status. Public Health Nutr. (2008) 11:897–904. 10.1017/S1368980007001231 18005487

[93] Saelens BE Epstein LH. Behavioral engineering of activity choice in obese children. Int J Obes Relat Metab Disord. (1998) 22:275–7. 10.1038/sj.ijo.0800570 9539197

[94] Laguna M Ruiz JR Lara MT Aznar S. Recommended levels of physical activity to avoid adiposity in Spanish children. Pediatr Obes. (2013) 8:62–9. 10.1111/j.2047-6310.2012.00086.x 22961693

[95] Patrick K Norman GJ Calfas KJ Sallis JF Zabinski MF Rupp J. Diet, physical activity, and sedentary behaviors as risk factors for overweight in adolescence. Arch Pediatr Adolesc Med. (2004) 158:385–90. 10.1001/archpedi.158.4.385 15066880

