# Supplementary figures and images for: Evaluating 12 Years of Implementing a Multidisciplinary Specialist Child and Adolescent Obesity Treatment Service: Patient-Level Outcomes
Source: Front Nutr. 2022 Jun 3;9:895091. doi: 10.3389/fnut.2022.895091 (PMC9204063; doi:10.3389/fnut.2022.895091)

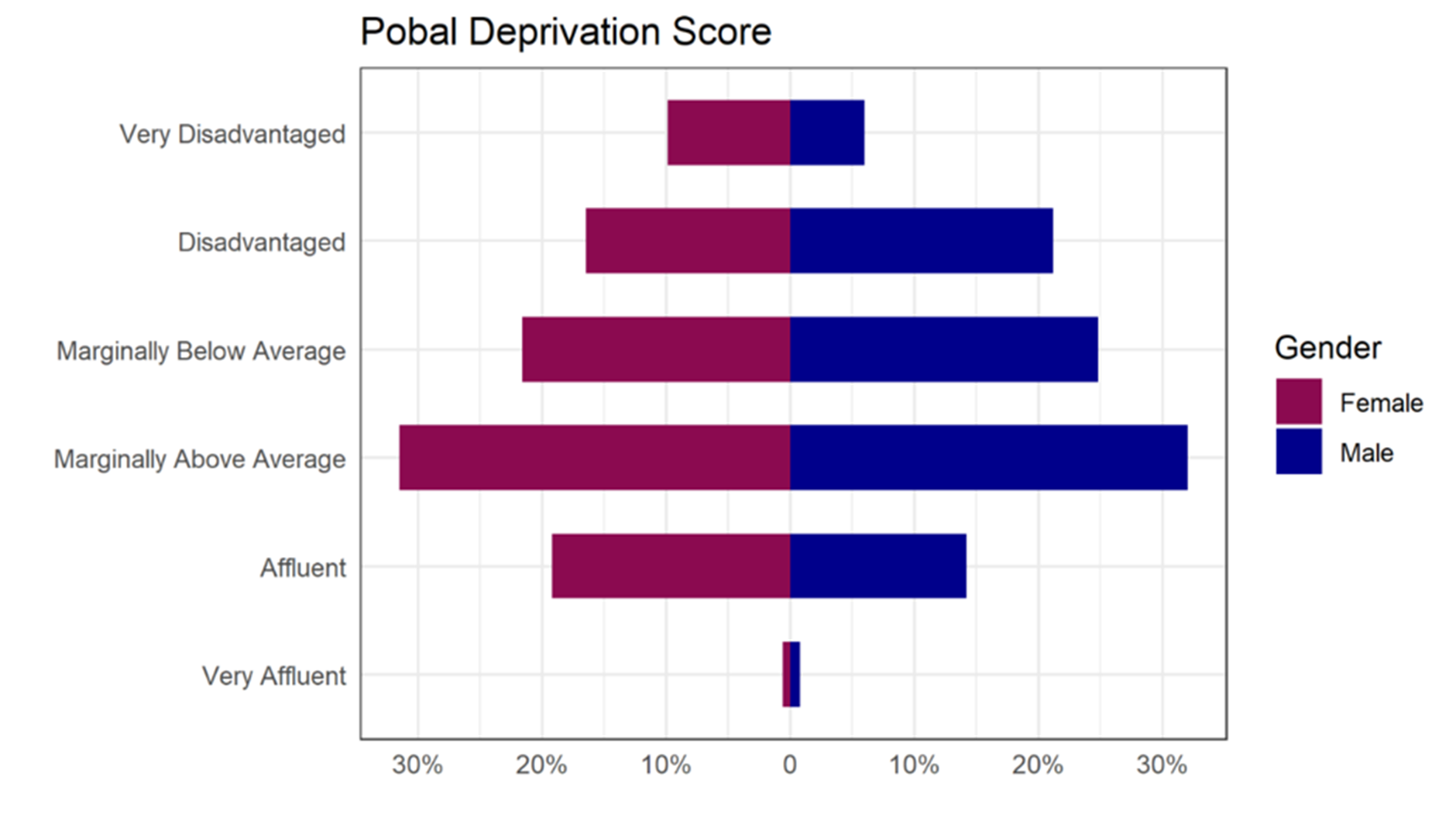

Supplement: Supplementary file 2 [file Image_2.PNG]

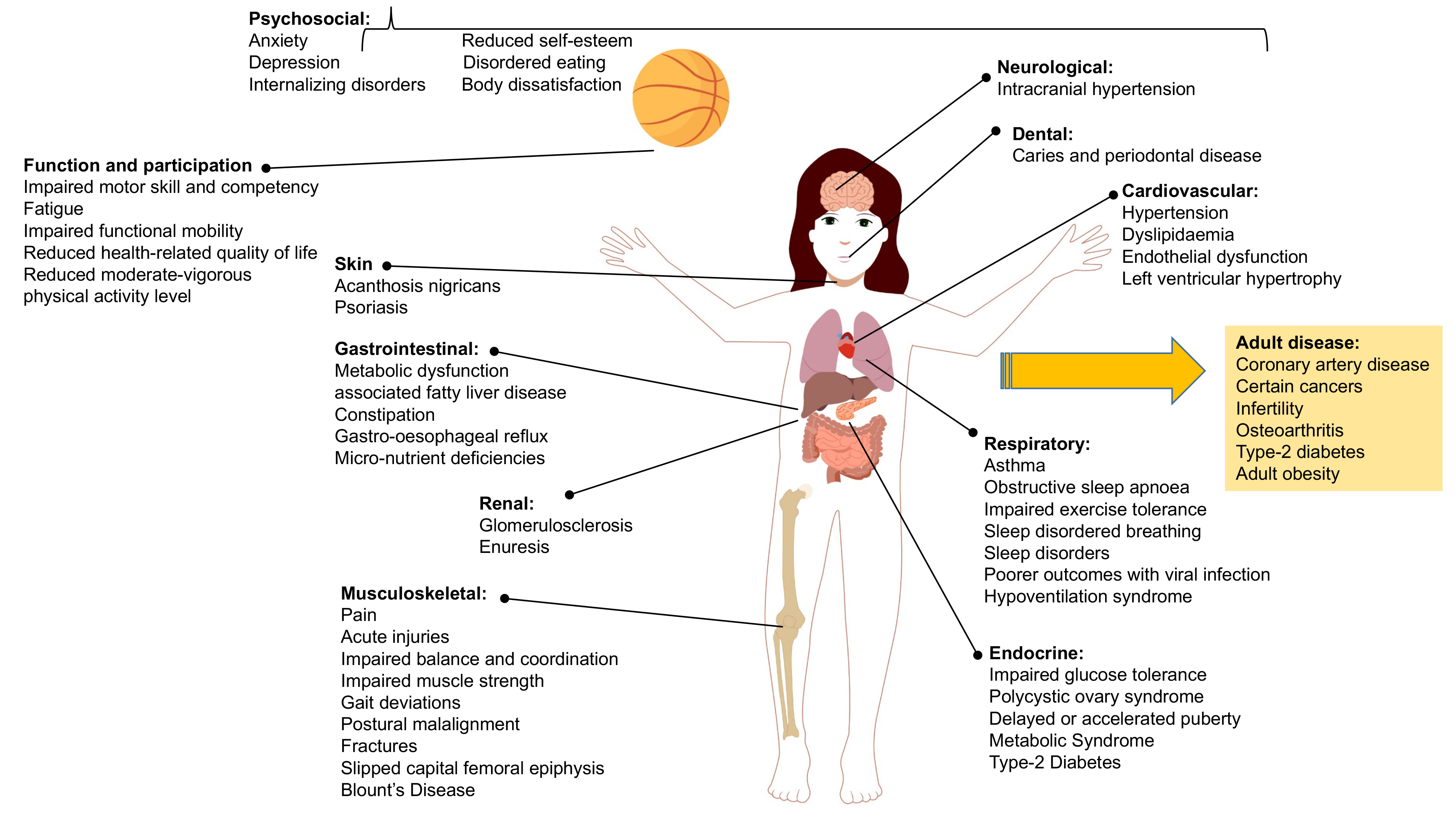

Supplement: Supplementary file 3 [file Data_Sheet_1.ZIP › Figure 1.tiff]
